# Nanopore sensing of individual transcription factors bound to DNA

**DOI:** 10.1038/srep11643

**Published:** 2015-06-25

**Authors:** Allison Squires, Evrim Atas, Amit Meller

**Affiliations:** 1 Department of Biomedical Engineering Boston University Boston, Massachusetts 02215 U.S.A.; 2 Department of Biomedical Engineering The Technion – Israel Institute of Technology Haifa, Israel, 32000

## Abstract

Transcription factor (TF)-DNA interactions are the primary control point in regulation of gene expression. Characterization of these interactions is essential for understanding genetic regulation of biological systems and developing novel therapies to treat cellular malfunctions. Solid-state nanopores are a highly versatile class of single-molecule sensors that can provide rich information about local properties of long charged biopolymers using the current blockage patterns generated during analyte translocation, and provide a novel platform for characterization of TF-DNA interactions. The DNA-binding domain of the TF Early Growth Response Protein 1 (EGR1), a prototypical zinc finger protein known as zif268, is used as a model system for this study. zif268 adopts two distinct bound conformations corresponding to specific and nonspecific binding, according to the local DNA sequence. Here we implement a solid-state nanopore platform for direct, label- and tether-free single-molecule detection of zif268 bound to DNA. We demonstrate detection of single zif268 TFs bound to DNA according to current blockage sublevels and duration of translocation through the nanopore. We further show that the nanopore can detect and discriminate both specific and nonspecific binding conformations of zif268 on DNA via the distinct current blockage patterns corresponding to each of these two known binding modes.

Transcription Factors (TFs) are the primary gene expression regulators in all known biological systems. Canonically, TFs operate by binding to their sequence-specific targets along genomic DNA, employing a complex search and binding mechanism that remains an intensive subject for research^1^. The TFs’ binding affinities and sequence specificity must be maintained within bounds that permit highly dynamic yet robust activation and deactivation of specific sets of genes during the cell cycle. Therefore, probing and characterizing TF binding to their DNA targets continues to be a major area of interest in molecular biology, and the implementation of novel experimental and computational approaches has drawn substantial focus. A wide host of techniques, both direct and indirect, commonly have been used to characterize sequence-specific protein binding to DNA, for example electrophoretic mobility shift assays (EMSA)^2^, inhibition of enzymatic degradation^3^, chromatin immunoprecipitation-based microarray^4^ or sequencing assays^5^, NMR^6^,^7^, x-ray crystallography^8^, bioinformatics^9^,^10^, and recently more advanced techniques such as optical tweezers^11^, AFM^12^, and even direct fluorescent visualization^13^, among others. However, most of these techniques require some combination of chemical cross linking of the TFs to the DNA, modification or tagging of the TF and DNA, and/or amplification assays, and may not have the capacity to resolve fine details of the TF/DNA complex, such as partial versus full binding of the TF domains to the DNA.

Solid-state nanopores are label- and tether-free, single-molecule biosensors for characterization of charged biopolymers, such as DNAs, RNAs, or proteins. An electrical field guides the biopolymers towards and into the nanopore, allowing them to probe individual molecules within an extremely dilute pool of analytes. Provided that the nanopore diameter is much smaller than the characteristic coil size, as each biopolymer passes through the pore, it must uncoil and thread through the narrow constriction in a linear fashion. Thus, the time-dependent ion conductance of the partially blocked pore can be directly related to local analyte characteristics as the nanopore scans the length of the translocating biopolymer.

Previous studies using solid-state nanopores focused on the detection of protein coatings on DNA, such as RecA^14^,^15^,^16^,^17^,^18^,^19^, or on individual large proteins such as streptavidin or antibodies bound to covalently-functionalized DNA^20^,^21^,^22^. Nanopore Force Spectroscopy (NFS)^23^ experiments have successfully employed voltage-driven removal of proteins from DNA to investigate the binding of restriction enzymes^24^,^25^, restriction endonucleases^26^, ligands for DNA aptamers^27^, and histones^28^,^29^,^30^ along DNA. Discrimination of non-protein changes in local analyte structure, for example in DNA structure^31^,^32^,^33^ or by adding PNA tags^34^, has also been demonstrated. In addition to many existing applications of nanopores, such as nucleic acid sequencing and barcoding^34^,^35^,^36^,^37^,^38^, protein characterization^22^,^39^,^40^,^41^,^42^,^43^,^44^,^45^,^46^ and detection of epigenetic modifications^47^,^48^,^49^, the ability to scan and identify features along the length of an analyte is particularly well-suited to determine the presence and binding interactions of protein-DNA complexes.

Due to their ability to rapidly scan hundreds of individual biopolymers, and resolve fine structural details along the analyte contour, solid-state nanopores have the potential to provide unique and rich information to facilitate our understanding of genetic regulation by TFs. However, to date the ability to detect small DNA-binding proteins reversibly bound to a specific recognition site along DNA, without the need for covalent functionalization or cross-linking, has proved elusive.

We demonstrate here that single-molecule measurements using solid-state nanopores can resolve single small proteins – the canonical zinc-finger DNA-binding domain of the Early Growth Response 1 (zif268) – bound natively to a recognition site. By performing single-molecule analysis of the ion current sublevels that emerge from the translocation of zif268/DNA complexes, we show that the nanopore can resolve and discriminate specific vs. nonspecific binding conformations of zif268, which produce distinct translocation current blockage patterns that correspond well to the known conformation and behavior of zif268 in both recognition (specific) and search (nonspecific) binding modes, respectively.

## zif268

**Figure 1:**
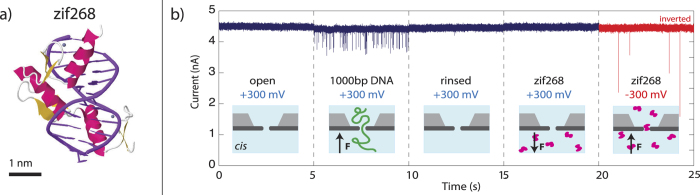
Continuous ion current traces of a 4 nm solid-state nanopore. **a**) Crystal structure of zif268 (PDB: 1AAY). **b**) Nanopore current at 300 mV bias, from left to right: Open pore current (4.5 nA); after the addition of 10 pM 1 kbp DNA to *cis* (transient blockages); clean after rinse with buffer (4.5 nA); after adding 10 nM zif268 (positively charged) to *cis* (no blockages); after reversing of the voltage to –300 mV (transient blockages).

The DNA-binding domain of Early Growth Response 1 (EGR1), a zinc finger protein known as zif268, was selected as a prototypical transcription factor. Zif268 binds tightly to DNA (0.2 nM < *k*_D_ < 5 nM), has been used extensively as a model system for studying how TFIIIA-like zinc fingers recognize DNA, and has even served as a basis for engineering several types of artificial DNA-binding proteins^50^. The crystal structure of zif268, solved via NMR by Pavletich^6^ and later improved upon by Elrod-Erickson^51^, is shown in Fig. 1a bound to DNA (PDB ID #1AAY)^52^. When bound, the three zinc fingers are situated in the major groove of the DNA and contact both bases and the phosphate backbone. The zinc fingers wrap ~180° around the DNA, forming a complex that is ~3 nm in diameter.

Figure 1b illustrates that the unbound zif268 does not interfere with normal operation of a small (~4 nm) solid state nanopore sensor. The pore is initially clean with a steady current of 4.5 nA at +300 mV bias (relative to *cis*). Upon addition of 1000 bp DNA to the *cis* chamber, transient current blockades indicate translocation of DNA through the nanopore. After a 10x volume rinse with buffer, no further translocations are observed, indicating that the DNA can be removed by dilution. As expected, addition of 10 nM of the positively charged zif268 (*p*I ≈ 10.5) to *cis* produces no translocations under +300 mV bias, while at −300 mV translocations are observed.

## Unbound zif268 and zif268_GST characterization

**Figure 2:**
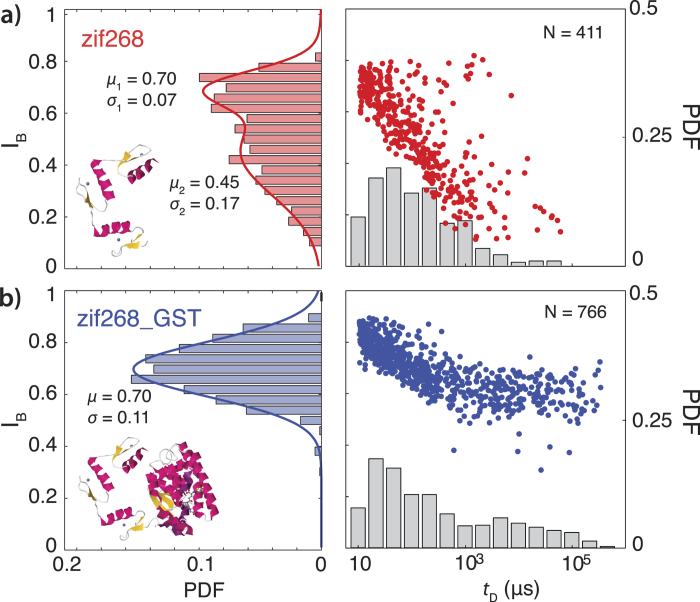
Translocations of unbound protein. **a**) zif268, showing two distinct blockage levels (*I*_*B*_ = 0.70 and *I*_*B*_ = 0.45) according to its probability density function (PDF), **b**) zif268_GST, showing only a single, broad blockage level (*I*_*B*_ = 0.70). Insets (left): PDB of zif268 and composite PDB of zif268 with GST. To facilitate visualization of population density, a random white noise offset below the acquisition rate of this data (–2 < ∆*t* <  + 2 μs, acquisition rate 250 kHz) has been added to the duration of each blockage level in the event diagram.

We began our study by characterization of the unbound zif268 (10.6 kDa) and the bulkier zif268_GST (37 kDa, GST purification tag left uncleaved) at negative bias. In Fig. 2 we display event diagrams (fractional blockade current, *I*_B_ = *i*_*blocked*_*/i*_*open*_ versus translocation dwell-times *t*_*D*_) as well as histograms of *I*_*B*_ and *t*_*D*_, of the two proteins. These results reveal a surprising pattern: while translocations of zif268 (*N* = 411) are relatively deep and exhibit at least two distinct blockage levels (Fig. 2a: *I*_*B*_ = 0.45 and 0.70), translocations of zif268_GST (*N* = 766) exhibit only a single blockage level (*I*_*B*_ = 0.70), as shown in Fig. 2b. This suggests that free zif268 can adopt at least geometrically distinct two conformations, while zif268_GST adopts one. One possible source of this difference is the charge on each domain: although both zif268 and zif268_GST both have net positive charge, each individual zinc finger is predicted to have a positive charge (*p*I = 8.8, 9.9, and 11.1 for ZF_1, ZF_2, and ZF_3, respectively, per EMBOSS calculation)^53^ while the GST is slightly negative at neutral *p*H (*p*I = 6.2). Thus the smaller but highly charged zif268 may adopt a more expanded conformation due to mutual repulsion of the three zinc fingers, while the oppositely charged domains of zif268_GST may tend to stick together in a more compact conformation with lower net charge that can explain the shift to longer times in *t*_*D*_^54^.

## Characterization of zif268 bound to DNA

**Figure 3:**
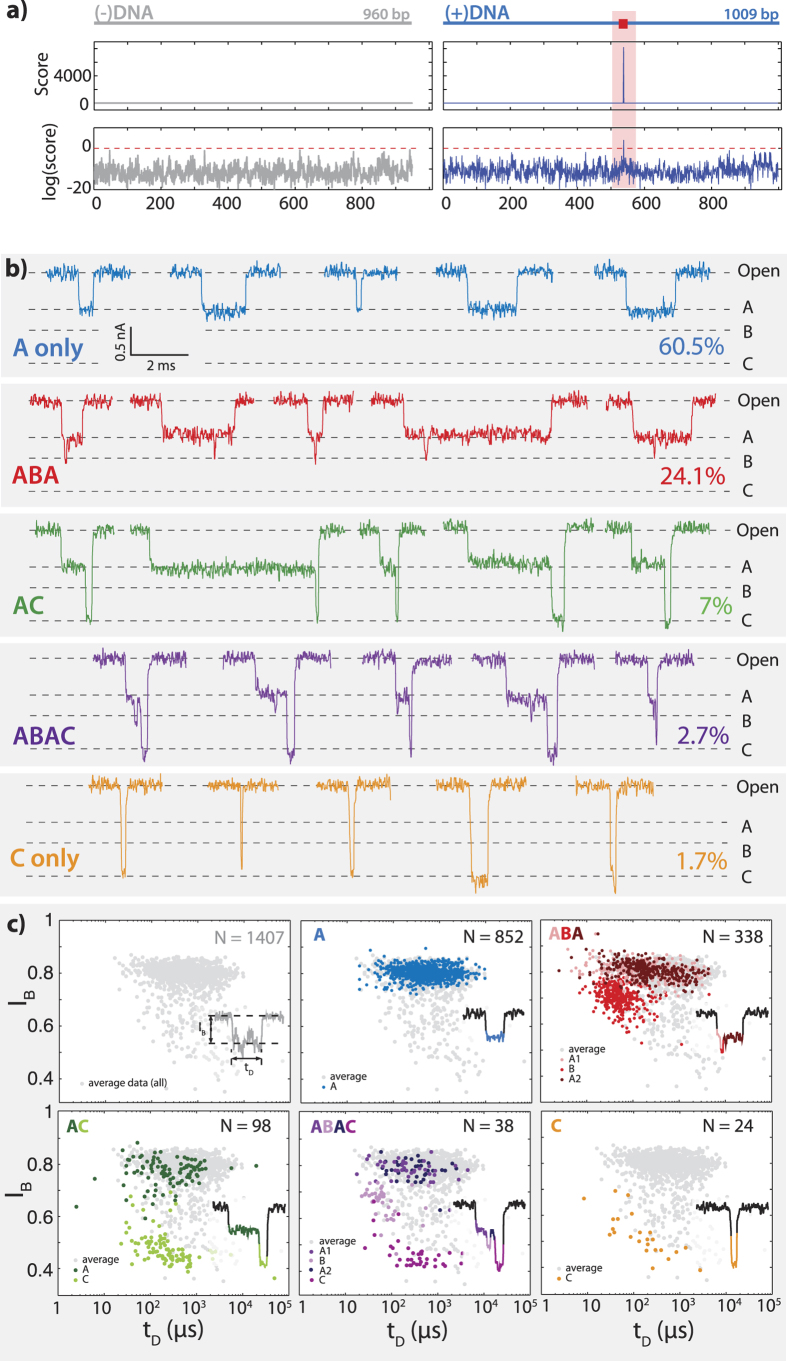
Blockage level patterns for zif268 + DNA in a thinned nanopore. **a**) DNA samples for binding: (−)DNA contains no binding sites and (+)DNA contains one centered binding site (red square), as identified by scoring the sequences according to a zif268 position weight matrix (see Supplementary Information for details, Table S1). **b**) DNA sample events showing all observed blockage sublevel patterns: A, ABA, AC, ABAC, and C. **c**) Scatter plots showing event average depth *I*_B_ and duration *t*_D_ (gray) as compared to sublevel depth *I*_B_^i^ and duration *t*_D_^i^ (by color, as indicated on inset sample events). To facilitate visualization of population density, a random white noise offset below the acquisition rate of this data (−2 < ∆*t* < + 2 μs, acquisition rate 250 kHz) has been added to the duration of each blockage level in the event diagram.

Translocation of bound zif268 may be observed in the fine structure of translocation events using smaller, thinned nanopores (*d* = 3.5 nm, effective thickness ~7 nm) for improved signal-to-noise ratio. Unlike the zif268_GST + DNA complexes (Supplementary Information, Figure S8), the small zif268 + DNA events exhibit a diverse behavior that may be classified into at least five distinct categories according to the observed current blockage pattern. Figure 3a shows schematically the design of two DNA samples used in this study, (–)DNA (left), which does not contain any predicted binding sites for zif268, and (+)DNA (right), for which one centered binding site for zif268 is predicted. These DNA fragments (960 bp and 1009 bp, respectively) were prepared by PCR amplification from the plasmid M13KO7 (NEB N0315S), with one centered binding site for zif268 (5’ GCGTGGGCG 3’), as detailed in the Supplementary Information. The location of the binding site is marked in Fig. 3a with a red square. Binding sites were predicted by scoring both sequences using a position weight binding matrix for zif268 (Transfac database 7.0)^55^,^56^. Prior to detection with a nanopore, DNA samples were pre-incubated at a ratio of 1:200 with purified recombinant zif268 (see Supplementary Information for details).

Figure 3b shows sample translocation events for (+)DNA incubated with zif268 (data using the (-)DNA and zif268 is shown in Figure S9). An analysis of our data suggests that three main blockage sublevels, A, B, and C, exist in the majority of events, and that these three levels appear in five distinct patterns rather than in random combinations. The five most frequently observed patterns are: A only (light blue, 60.5%), ABA (red, 24.1%), AC (green, 7%), ABAC (purple, 2.7%) and C only (gold, 1.7%). The remaining 4% of events in this data set represent a wide variety of blockage patterns, none of which account for more than 1% of the total translocation events observed. Blockage level patterns were determined using a maximum-likelihood level-finding algorithm to define an initial set of many possible sublevels. These sublevels were grouped into three major populations, representing the A, B, and C sublevels, which could be separated by selecting thresholds between them. The initial sublevels were then used to reconstruct the final event pattern by combining any neighboring sublevels that had not crossed one of these thresholds (see Supplementary Information for details).

Figure 3c shows event diagrams for the dwell time *t*_*D*_ and blockage level *I*_*B*_ associated with the sublevels for events exhibiting one of the five translocation patterns (A: blue, ABA: red, AC: green, ABAC: purple, C: orange). Each colored point represents a sublevel blockage level, *I*_*B*_^*i*^ , and its corresponding sublevel dwell time, *t*_*D*_^*i*^, where the superscript *i* denotes one of the three sublevels (A, B, or C). The background gray scatter plot shown in all frames and at top left represents the total dwell time *t*_*D*_ and mean blockage level *I*_B_ for all observed translocations. The large number of events with unusually deep blockage sub-levels suggests additional observed patterns in the DNA-TFs complexes as compared to bare DNA, which typically displays a single blockage level in small nanopores which exclude folded DNA translocation^57^.

**Figure 4:**
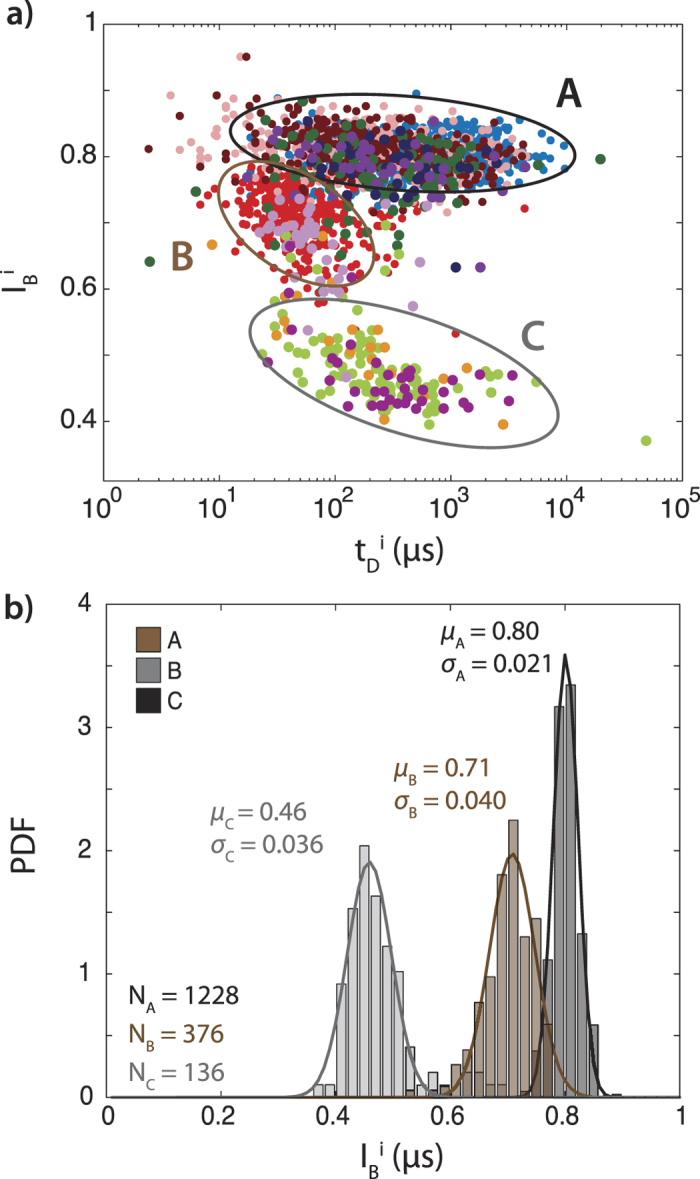
Cumulative A, B, and C sublevels from all categorized blockage patterns. **a**) Superimposed scatter plot of A, B, and C sublevels from all blockage patterns. Ellipses indicate A, B, and C populations. **b**) Blockage level distributions for the three sublevels across all blockage patterns.

In Fig. 4 we further analyze our classification of the translocation event types by examining sublevel properties across the entire data set. Figure 4a is a superposition of all event diagrams from Fig. 3b (colors consistent with Fig. 3b), and demonstrates that the A, B, and C sublevels from different translocation patterns also overlap in their durations, forming three distinct populations, highlighted here with ovals. For example, although both ABA:(*t*_D_^A1^, *I*_B_^A1^) sublevels (pink) and ABA:(*t*_D_^A2^, *I*_B_^A2^) sublevels (maroon) mostly coincide with the event-averaged current level and dwell time, ABA:(*t*_D_^B^, *I*_B_^B^) sublevels (red) form a new cluster at short dwell times *t*_D_^B^ = 50 μs and deeper blockage *I*_B_^B^ = 0.71. Similarly, the AC (green) sub levels (*t*_D_^C^, *I*_B_^C^) forms a separate cluster at moderate *t*_D_^C^ (256 μs) and deep *I*_B_^C^ = 0.46, which are not otherwise observable in the event-averaged analysis. When superimposed as shown in Fig. 4a, it is clear that the clusters formed for the B and C sublevels exhibit consistent timing and blockage level across all five event patterns, suggesting that these sublevels represent three different states and/or geometries of the analyte molecule that arise during translocation. This conclusion is further illustrated by plotting the histograms of each group of sublevels (Fig. 4b), across all observed patterns, which clearly show three distinct current blockage sublevels.

**Figure 5:**
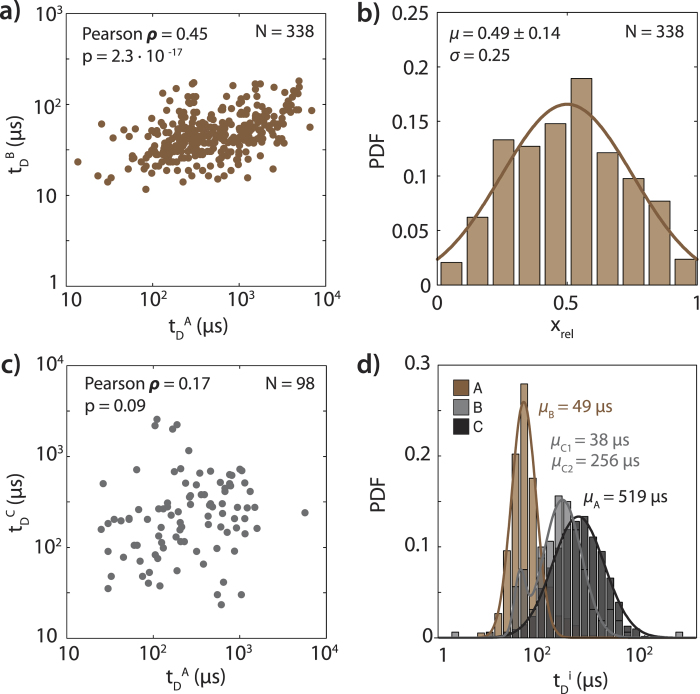
Timing of A, B, and C sublevels. **a**) Correlation of B level duration to total A level duration in ABA events (Pearson ***ρ*** = 0.45 correlation, with significant p = 2.3×10^−17^). **b**) Distribution of observed relative position *x*_rel_ of the B level within ABA events with Gaussian fit, N = 338. **c**) Correlation of C level duration to A level duration in AC events (Pearson ***ρ*** = 0.17 correlation, with non-significant p = 0.09). **d**) Distribution of observed durations of A, B, and C levels from all classifiable blockage level patterns observed.

In order to interpret our results shown in Fig. 3 and Fig. 4, we further investigated the sublevel dwell-times. It is evident that the A blockage level may be attributed to the translocation of double-stranded DNA without bound protein (Supplementary Information, Figure S8 and Figure S9). But what do the B and C sublevels represent? The nanopore should be able to accommodate translocation of the bound zif268 + DNA, and the binding site is located at the center of the DNA. It is therefore reasonable to hypothesize that passage of the complex through the nanopore would create a transient extra blockage such as B sublevel during the course of an event. As shown in Fig. 5a, the duration of the B sublevel is significantly correlated to the total dwell time of the DNA only sublevels (*t*_D_^A^ = *t*_D_^A1^ + *t*_D_^A2^) with a Pearson correlation coefficient *ρ* = 0.45 (high significance, two-tailed *p* = 2.3 × 10^−17^ relative to null). Moreover, the timing of the B sublevel within the overall event is consistent with a single binding site at the center of the dsDNA. Figure 5b shows the relative position of the B sublevel within the event, where *x*_rel_ = *t*_B_center_/(*t*_D_^A1^ + *t*_D_^A2^) so that *x*_rel_ = 0 corresponds to a B sublevel at the start of the event, and *x*_rel_ = 1 corresponds to a B sublevel at the end of the translocation event. The binding site on the sample described here is located at *x*_rel_ = 0.55 within the 1009 bp double-stranded (+)DNA. We find an expected value of <*x*_rel_> = 0.49 ± 0.14, which places the actual binding site well within our estimation, with an effective position error of ±140 bp for this sample. In the case of (−)DNA + zif268, the B sublevels, and most notably the ABA pattern (Figure S9), are much less frequent as compared with the (+)DNA + zif268 (4.3% and 11.5%, respectively). Additionally as shown in Figure S11, in the (−)DNA + zif268 case the B sublevels are not found in the center; and rather fall into two populations at *x*_rel_ = 0.49 ± 0.016 and *x*_rel_ = 0.79 ± 0.014, perhaps suggesting the existence of one or more preferred binding sites, not predicted by the binding models, for zif268 located off-center along the (−)DNA.

In contrast to the B sublevel, the duration of the C sublevel is *not* significantly correlated to the DNA dwell time *t*_D_^A^ (Fig. 5c), with a Pearson correlation coefficient *ρ* = 0.17, which is not statistically significant at 95% (two-tailed *p* = 0.09 relative to null). Based on Fig. 5a, the dwell time of the section of the analyte that causes the B sublevel is related to the overall velocity of the DNA through the nanopore, and is preceded and followed by the passage of bare DNA (A sublevels). These evidences are consistent with passage of a bound zif268 through the nanopore, located roughly at the predicted binding site (Fig. 5b). However, because the duration of the C sublevel is weakly related to the velocity of the DNA, and occurs after the DNA has passed mostly through the pore, we can conclude that the state of the analyte resulting in the C sublevel is substantially different from that involved in the ABA events.

Figure 5d shows log scale histograms of the duration of sublevels A, B, and C across all types of recorded events. The B sublevels have short dwell times (49 μs) compared to both the A and C sublevels (519 and 256 μs, respectively), which is consistent with a bound complex with a short footprint (<10 bp) passing through the nanopore on a long (1 kbp) piece of DNA. However, if the bound complex were to travel roughly at the same velocity as the rest of the DNA, then the expected dwell time for the B sublevel would be 5 μs, a timescale that is shorter than our current sensing capability. This raises the possibility that a fraction of actual ABA events are misclassified as A events (bare DNA) because the B sublevel is too short to be detected. This observation may explain the relatively small fraction of the ABA events (roughly 24%) and relatively large fraction of A events (61%), whereas according to EMSA (200:1 protein:DNA incubation ratio) nearly 100% of the DNA used in this study should have one specifically bound zif268 at center (Figure S6 and Figure S7).

But what is the source of the C sublevel observed at the end of many events, and is it related to zif268 binding? A direct comparison of bare DNA with (−)DNA + zif268 and (+)DNA + zif268 shown in Figure S9 also reveals that: 1) patterns including B or C sublevels are very rarely detected for bare DNA, and 2) C sublevels, and most notably the AC pattern, are detected with similar frequency for both (−)DNA + zif268 and (+)DNA + zif268 samples (15.8% and 13.9%, respectively). Recently published findings on a zif268 conformational switch from its specific to non-specific bound forms on DNA suggest that we should indeed expect to see two very different conformations – and therefore different blockage levels – for zif268 +DNA translocations. Zandarashvili and co-workers present NMR data that reveals fast, highly dynamic motions of the zinc finger 1 (ZF1) domain of zif268 away from the duplex DNA when the transcription factor is non-specifically bound to DNA^58^, and that in this state zif268 slides easily and quickly along DNA. In its bound recognition state, zif268 takes on the structure previously determined by Elrod-Erickson, Pavletich, and Pabo^6^,^51^, where all three zinc fingers are bound tightly in the major groove of the DNA. From studies of the kinetics of zif268’s movement between nonspecific DNA duplexes, Zandarashvili *et al.* also infer that the constant motion of ZF1 and its position lifted away from the DNA duplex facilitates intersegment transfer of the transcription factor between distant regions of DNA, speeding up the binding site search process^58^. The timescales associated with the larger, nonspecific binding conformation also provide key clues to the expected blockage pattern: In “search” mode, the zif268 zinc finger 1 explores its expanded conformation on a nanosecond timescale, and the zif268 slides along DNA with a microsecond timescale (in the absence of force), both of which occur much faster than typical translocation speeds^58^.

**Figure 6:**
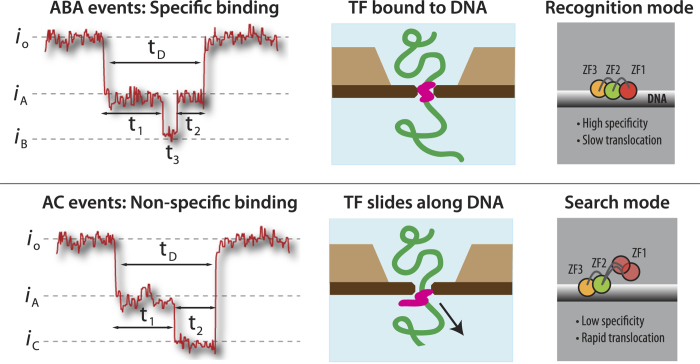
Suggested model and interpretation of results. Left: Diagram of blockage levels and timing for ABA and AC events. Center: Cartoon representation of suggested mechanism. Right: Recognition mode (ABA) and search mode (AC) conformations, as determined by Zandarashvilli *et al.*

These known conformations allow us to more clearly identify the sublevels and observed blockage patterns for zif268 +DNA translocations, as depicted in Fig. 6: We propose that the B sublevel represents translocation of the bound zif268 +DNA complex, while the C sublevel represents removal of nonspecifically bound zif268 from the DNA. The short duration and extra blockade of the B sublevel compared to bare DNA blockage level are both consistent with the temporary presence in the sensing volume of a locally bulky section of DNA, which is precisely the description of zif268 bound to a 9 bp region of DNA. Similarly, the deep blockage of the C level is consistent with the larger “search” conformation, while its presence at the end of events is consistent with its ability to slide easily along DNA.

The translocation of unbound zif268 described earlier in this paper further supports this possibility; we note that the untagged zif268 protein exhibited deep blockages and multiple conformations (resulting in the broad *I*_*B*_ distribution, Fig. 2). This also implies that if zif268 is responsible for the C sublevel, its conformation when creating this blockage must be more similar to its conformation during free translocation than to its specifically bound conformation in the major groove of DNA, which agrees with the “search” conformation established by Zandarashvilli *et al.* This expanded conformation would also enhance any potential interaction with the nanopore walls, potentially aiding shear-induced rupture of the zif268 from the DNA. The electrophoretic force applied on double-stranded DNA threaded in a pore can reach ~10^2^ pN at 300 mV (depending on various variables, such as the effective charge of the DNA and interactions with the pore walls) which is comparable to the force required to rupture zif268 off of DNA as measured by AFM^12^,^23^. We note, however, that the rupture force depends strongly on the loading rate and directionality, which require further investigation for the nanopore system.

## Summary and Discussion

The ability to detect the presence of bound complex, determine the protein conformation, and optionally remove transcription factors from single molecules of DNA opens a wide range of possibilities for nanopore characterization of transcription factors bound to DNA. In our study, the combination of high salt and the high protein:DNA ratio employed in this experiment enabled concurrent observation of both specific and nonspecific binding modes. Solid-state nanopores are chemically robust, so this technique could be extended to a wide range of binding and detection conditions.

Our results indicate that nanopore sensors can not only identify the presence of single small transcription factors bound to DNA, but that they can detect and discriminate between specific and nonspecific binding modes. The observed blockage patterns associated with translocation of bound zif268 +DNA correspond well to the established recognition and search binding modes. These results highlight the rich data available upon examining the fine structure of blocked current sublevels within a single translocation event. This unique technique provides access to entirely new types of single-molecule information about DNA binding to transcription factors.

## Methods

zif268 (courtesy of Scot Wolfe, University of Massachusetts Medical School) was cloned into a pGex2t plasmid (GE Healthcare Life Sciences) along with a cleavable glutathione S-transferase (GST) tag. Fusion protein was expressed in BL21 cells grown in LB media at 37 °C, and purified using a glutathione sepharose column (GE Healthcare Life Sciences). GST tags were cleaved from zif268 via room-temperature overnight incubation with thrombin (GE Healthcare Life Sciences) and then eluted. Fusion GST_zif268 was eluted from the purification column using excess glutathione. Purified protein (stock concentration 50 μM) was aliquoted and stored at −80 °C until use.

Specific binding of both zif268_GST and zif268 to DNA was first verified using 50 bp oligomers for EMSA. Both zif268_GST and zif268 show similar binding affinity for the wild-type binding site, GCGTGGGCG (Supplementary Information, Figure S4). The DNA used in nanopores experiments consists of a 1000 bp sequence containing a single, centered wild-type binding site, PCR-amplified from a commercially available plasmid (M13KO7). A 1000 bp negative control without this binding site, as defined by a maximum variation of two substitutions, deletions, or insertions as compared to the consensus binding site, was computationally identified and PCR-amplified (from λ-DNA, see Supplementary Information for details, Figure S3). All PCR primer sequences and amplified sequence details are available in the Supplementary Information. Binding affinity for the specific and nonspecific sites on the 1000 bp DNA samples in the nanopore buffer conditions (1M KCl) was verified by EMSA (Supplementary Information, Figure S6). All binding was performed immediately prior to nanopore experiments, with a 30 min incubation of 200:1 protein:DNA.

Nanopores were fabricated in freestanding low-stress amorphous silicon nitride (SiN_x_) membranes, deposited using Low Pressure Chemical Vapor Deposition (LPCVD) to a thickness of either 25 nm (DNA + zif268_GST experiments) or 60 nm (DNA + zif268 experiments) on <100> 350 μm thick silicon wafers. The 60 nm thick membranes were subsequently locally thinned to improve signal-to-noise ratio^46^ by controlled Reactive Ion Etching (RIE) in ~1.5 μm diameter circular regions patterned by full-wafer optical lithography, leaving ~15 nm thick wells in which pores were later fabricated (see Supplementary Information). Nanopores were drilled using a JEOL 2010F transmission electron microscope as previously described^59^. Nanopores were cleaned using piranha and assembled in a custom Teflon cell, and low-noise electrical measurements were performed in a custom-built electrically shielded setup using Ag/AgCl electrodes and an Axopatch 200B patch-clamp amplifier, all as previously described^34^,^37^,^57^,^60^. All measurements were performed in 1M KCl, 10 mM Tris, pH 7.5.

The current flowing through the nanopore under the applied bias was measured using two homemade Ag/AgCl electrodes connected to an Axon 200 amplifier and sampled at 250 kHz/16 bits using a DAQ card (PCI-6534, National Instruments). Custom LabVIEW software was used to detect/save events and control the voltage applied across the pore. Event classification was performed off-line using custom Matlab code to identify and separate current blockage sublevels.

## Additional Information

**How to cite this article**: Squires, A. *et al.* Nanopore sensing of individual transcription factors bound to DNA. *Sci. Rep.* **5**, 11643; doi: 10.1038/srep11643 (2015).

## Supplementary Material

Supplementary Information
