# Supplementary material for: Nanopore sensing of individual transcription factors bound to DNA
Source: Sci Rep. 2015 Jun 25;5:11643. doi: 10.1038/srep11643 (PMC4479991; doi:10.1038/srep11643)
Supplement: Supplementary Information [file srep11643-s1.pdf]

Supporting Information for:

Nanopore sensing of individual transcription factors bound to DNA

Allison Squires[1], Evrim Atas[1], and Amit Meller*[1,2]

[1]Department of Biomedical Engineering
Boston University
Boston, Massachusetts 02215
U.S.A.

[2]Department of Biomedical Engineering
The Technion – Israel Institute of Technology
Haifa, Israel 32000

*Corresponding author. E-mail ameller@bm.technion.ac.il

Table of Content

1. Expression and purification of zif268.
2. Zif268 plasmid and sequence.
3. Target DNA sample design.
4. Target DNA PCR.
5. Binding verification assay: zif268 + DNA.
6. Bound DNA + zif268_GST Translocations.
7. Translocation events sublevel analysis.
8. Additional data for DNA + zif268 translocations
9. References.

1. Expression and purification of zif268

The zif268 (courtesy of Scot Wolfe, University of Massachusetts Medical School) was cloned into a pGex2t plasmid (GE Healthcare Life Sciences) directly following a glutathione S-transferase (GST) tag and thrombin cleavage site for simplified purification. Because of its bulk, the GST tag must be cleaved off to allow translocation of the DNA-bound complex in a 4-5 nm pore, but is beneficial when the desired outcome is removal of the protein by the nanopore. Fused zif268_GST is expressed in BL21 competent cells grown in 2xYT media with ampicillin at 37°C, induced with IPTG. Cells are harvested and lysed in a buffer containing glycerol, β-mercaptoethanol (βME), nucleases and protease inhibitor but no EDTA. The lysate is incubated at 4°C with glutathione sepharose fast-flow resin (GE Healthcare) and washed on a gravity-flow column. The wash buffer contains 1xPBS with 20% glycerol, 160 mM additional KCl (total ionic strength ~300 mM, similar to nanopore conditions) and 1 mM βME. The fused zif268_GST product was eluted at this point using excess glutathione in a buffer containing glycerol, βME, and 200 mM KCl. Figure S1 shows a Coomassie stained denaturing gel (SDS-PAGE) of the elutions for the most recent batch of this product. The fused zif268_GST was also analyzed against a protein size standard using a 116 BioRad Pro260 system to determine yield (3 mg, stock 100 μM) and purity (~90%). The uncleaved product has a total molecular weight of 37 kDa (27 kDa for GST, 10 kDa for zif268).

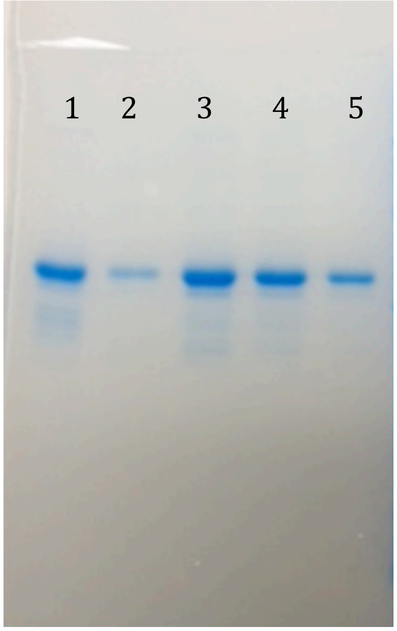

Figure S1: SDS-PAGE of zif268_GST purified protein, prior to cleavage with thrombin. Coomassie stain. Lane 1: Protein standard (100 ng/μl). Lane 2: Elution 1. Lane 3: Elution 2. Lane 4: Elution 3. Lane 5: Elution 4. Highly purified protein was obtained ~100 μM, 3 mL zif268_GST

To obtain cleaved zif268 for nanopore binding site mapping, bovine thrombin (Sigma) was added on-column to a separate expression batch and left to cleave overnight at room temperature. Purified zif268 was eluted and cleavage halted using PMSF. Figure S2 shows a Coomassie-stained denaturing gel (SDS-PAGE) of the elutions for the most recent batch of this product. The cleaved product was also analyzed against a protein size standard using a BioRad Pro260 system to determine yield (20 mg, stock 50 μM) and purity (~85%). The primary source of impurities is the lighter weight band, which we believe to be single zinc finger products. Note that the expected cleaved product has a total molecular weight of 10 kDa. Stock for both fused zif268_GST and cleaved zif268 was aliquoted and stored in 20% glycerol at -80°C.

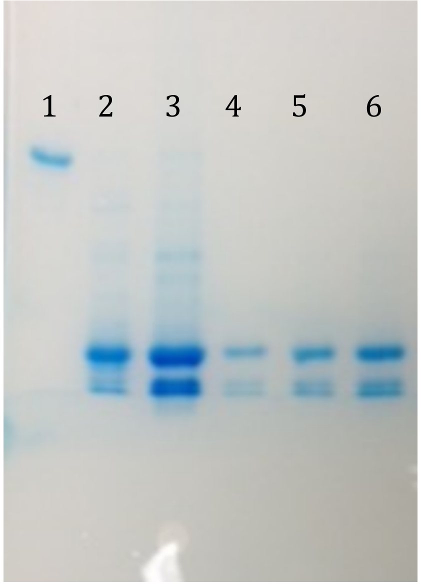

Figure S2: SDS-PAGE of cleaved zif268 purified protein with coomassie stain. Lane 1: protein standard (50ng/μl). Lane 2: Elution 1, Lane 3: Elution 2. Lane 4: Elution 3. Lane 5: Elution 4. Lane 6: Pure protein obtained after purification; ~ 100 μm, 3 mL zif268_GST.

2. Zif268 plasmid and sequence

Sequence of zif268 in pGex2t vector

Personal communication from Scot Wolfe (University of Massachusetts Medical School)
CAPS = fusion protein (GST + zif268)
UNDERLINE = thrombin recognition site
// = thrombin cleavage site
RED CAPS = zif268 (Homo sapiens)

Bp # 1-1491
acgttatcgactgcacggtgcaccaatgcttctggcgtcaggcagccatcggaagctgtggtatggctgtgcaggtcgtaaatcactgcataattcgtgtcgctcaaggcg
cactcccgttctggataatgttttttgcgccgacatcataacggttctggcaaatattctgaaatgagctgttgacaattaatcatcggctcgtataatgtgtggaattgtgagcg
gataacaatttcacacaggaaacagtattcATGTCCCCTATACTAGGTTATTGGAAAATTAAGGGCCTTGTGCAACCCAC
TCGACTTCTTTTGGAATATCTTGAAGAAAAATATGAAGAGCATTTGTATGAGCGCGATGAAGGTGATA
AATGGCGAAACAAAAAGTTTGAATTGGGTTTGGAGTTTCCCAATCTTCCTTATTATATTGATGGTGATG
TTAAATTAACACAGTCTATGGCCATCATACGTTATATAGCTGACAAGCACAACATGTTGGGTGGTTGTC
CAAAAGAGCGTGCAGAGATTTCAATGCTTGAAGGAGCGGTTTTGGATATTAGATACGGTGTTTCGAGA
ATTGCATATAGTAAAGACTTTGAAACTCTCAAAGTTGATTTTCTTAGCAAGCTACCTGAAATGCTGAAA
ATGTTCGAAGATCGTTTATGTCATAAAACATATTTAAATGGTGATCATGTAACCCATCCTGACTTCATG
TTGTATGACGCTCTTGATGTTGTTTTATACATGGACCCAATGTGCCTGGATGCGTTCCCAAAATTAGTT
TGTTTTAAAAAACGTATTGAAGCTATCCCACAAATTGATAAGTACTTGAAATCCAGCAAGTATATAGC
ATGGCCTTTGCAGGGCTGGCAAGCCACGTTTGGTGGTGGCGACCATCCTCCAAAATCGGATCTGGTTC
CGCGT//GGATCC**GAACGCCCATATGCTTGCCCTGTCGAGTCCTGCGATCGCCGCTTTTCTCGCTC**
GGATGAGCTTACCCGCCATATCCGCATCCACACAGGCCAGAAGCCCTTCCAGTGTCGAATCTGC
ATGCGTAACTTCAGTCGTAGTGACCACCTTACCACCCACATCCGCACCCACACAGGCGAGAAGC
CTTTTGCCTGTGACATTTGTGGGAGGAAGTTTGCCAGGAGTGATGAACGCAAGAGGCATACCAA
AATCCATTTAAGACAGAAGGACTGAcaattcatcgtgactgactgacgatctgcctcgcgcgtttcggtgatgacggtgaaaacctctgacac
atgcagctcccggagacggtcacagcttgtctgtaagcggatgccgggagcagacaagcccgtcagggcgcgtcagcgggtgttggcgggtgtcggggcgcagcc
atgacccagtcacgtagcgatagcggagtgtataattcttgaagacgaaagggcctcgtgatacgcctatttttataggttaatgtcatgataataatggtttcttagacgtc

3. Target DNA sample design

Zif268 consensus binding site: **5' – GCGG/TGGGCG – 3'[1].**

Table S1: Positional weight matrix for zif268[2]:

Position	A	C	G	T	
01	45.5	6.1	6.1	42.4	W
02	5.1	2.6	5.1	87.2	T
03	7.3	0.0	92.7	0.0	**G**
04	0.0	98.2	0.0	1.8	**C**
05	1.8	0.0	98.2	0.0	**G**
06	0.0	1.8	14.5	83.6	**T**
07	30.9	0.0	69.1	0.0	**G**
08	0.0	0.0	100.0	0.0	**G**
09	0.0	0.0	100.0	0.0	**G**
10	12.7	76.4	0.0	10.9	C
11	0.0	0.0	100.0	0.0	G

To demonstrate specific binding of both zif268_GST and the zif268, dsDNA oligomers were designed both with and without this binding site. Potential binding sites are defined by a minimum log score of 0 using the PWM and scoring criteria after Pfenning et al[3]. The scoring matrix *S* is defined as:

$$S_{ij} = \log\left(\frac{PWM_{ij} + b}{\sum_k PWM_{kj} + 4b}\right)$$

Dinucleotide frequencies were used to calculate background sequence probabilities. The score at each position was defined by:

$$\log(Score(n)) = \log\left(\frac{P_{\text{model}}(n)}{P_{\text{back ground}}(n)}\right)$$

The ~35 bp oligos in Figure S3a and the fluorescently labeled oligo in Figure S3b were used for EMSAs to verify binding. Longer DNA samples are necessary for use in nanopores, so ~1009 bp samples were designed using the criteria described above.

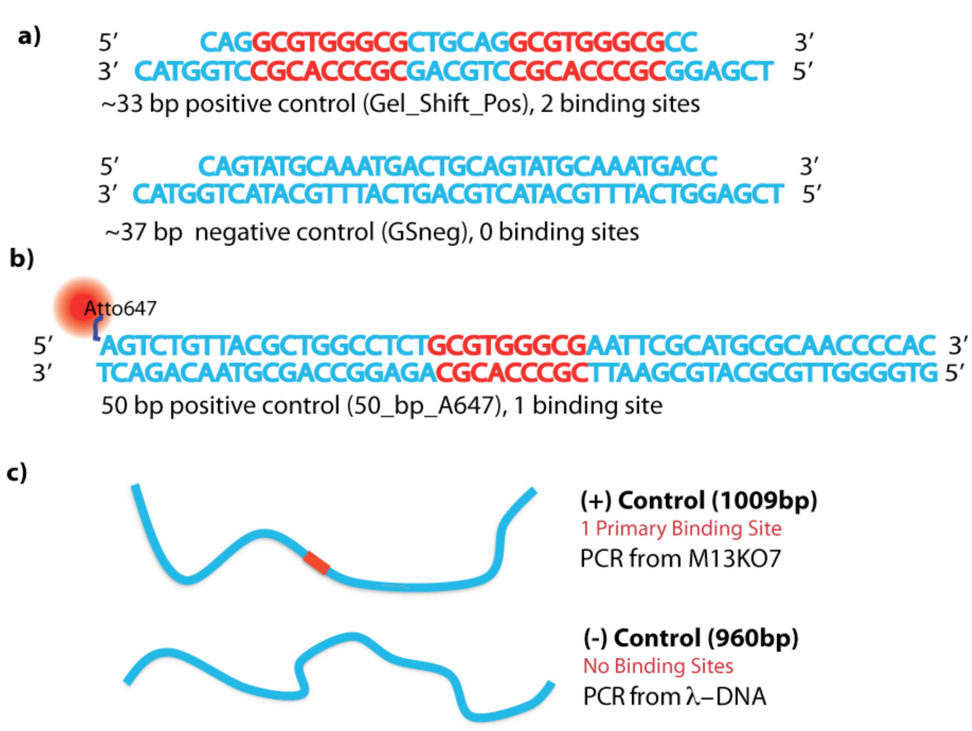

Figure S3: DNA samples for zif268 binding studies. a) Sequence of DNA oligomers to test binding of zif268 and zif268_GST to the consensus sequence. b) An Atto-647-labeled 50 bp oligo containing one binding site. c) Design of ~1000bp DNA sequences amplified from λ-phage and plasmid DNA with and without the consensus binding sequence.

4. Target DNA PCR

Target DNAs with and without a zif268 binding site were PCR-amplified from a commercially available material (M13KO7 plasmid and λ-DNA, respectively). PCR primer sequences and amplified sequence details are shown here:

(-) DNA: 960 bp DNA PCR-ed from lambda DNA (NEB), with no binding sites for zif268.
Primers:

forward 5’ GACATTACAAACGTCCTTCTC

reverse 5’ CATTACTCACTTCCCCACT

(-)DNA Sequence:
5’gacattacaaacgtccttctcggtgcatgccactgttgccaatgacctgcctaggaattggttagcaagttactaccggattttgtaaaaacagccctcctcatataaaaagtat
tcgttcacttccgataagcgtcgtaattttctatctttcatcatattctagatccctctgaaaaaatcttccgagtttgctaggcactgatacataactcttttccaataattggggaagtc
attcaaatctataataggtttcagatttgcttcaataaattctgactgtagctgctgaaacgttgcggttgaactatatttccttataacttttacgaaagagtttctttgagtaatcacttc
actcaagtgcttccctgcctccaaacgatacctgttagcaatatttaatagcttgaaatgatgaagagctctgtgtttgtcttcctgcctccagttcgccgggcattcaacataaaaa
ctgatagcacccggagttccggaaacgaaatttgcatatacccattgctcacgaaaaaaaatgtccttgtcgatatagggatgaatcgcttggtgtacctcatctactgcgaaaa
cttgacctttctctcccatattgcagtcgcggcacgatggaactaaattaataggcatcaccgaaaattcaggataatgtgcaataggaagaaaatgatctatatttttgtctgtcct
atatcaccacaaaatggacatttttcacctgatgaaacaagcatgtcatcgtaatatgttctagcgggtttgtttttatctcggagattattttcataaagcttttctaatttaacctttgtc
aggttaccaactactaaggttgtaggctcaagagggtgtgtcctgtcgtaggtaaataactgacctgtcgagcttaatattctatattgttgttctttctgcaaaaaagtggggaagt
gagtaatg

(+) DNA: 1009 bp DNA PCR-ed from the plasmid M13KO7, with one centered binding site for zif268 (GCGTGGGCG).

Primers:

forward 5’ CGTCAGGGCAAGCCTTATT

reverse 5’ ACCGTACTCAGGAGGTTTAGT

(+)DNA Sequence:
5’cgtcagggcaagccttattcactgaatgagcagctttgttacgttgatttgggtaatgaatatccggttcttgtcaagattactcttgatgaaggtcagccagcctatgcgcctg
gtctgtacaccgttcatctgtcctctttcaaagttggtcagttcggttcccttatgattgaccgtctgcgcctcgttccggctaagtaacatggagcaggtcgcggatttcgacacaa
tttatcaggcgatgatacaaatctccgttgtactttgtttcgcgcttggtataatcgctgggggtcaaagatgagtgttttagtgtattctttcgcctctttcgttttaggttggtgccttc
gtagtggcattacgtattttacccgtttaatggaaacttcctcatgaaaaagtctttagtcctcaaagcctctgtagccgttgctaccctcgttccgatgctgtctttcgctgctgagg
gtgacgatcccgcaaaagcggcctttaactccctgcaagcctcagcgaccgaatatatcggttatgcgtgggcgatggttgttgtcattgtcggcgcaactatcggtatcaagct
gtttaagaaattcacctcgaaagcaagctgataaaccgatacaattaaaggctccttttggagcctttttttttggagattttcaacgtgaaaaaattattattcgcaattcctttagttgt
tcctttctattctcactccgctgaaactgttgaaagttgtttagcaaaaccccatacagaaaattcatttactaacgtctggaaagacgacaaaactttagatcgttacgctaactatg
agggttgtctgtggaatgctacaggcgttgtagtttgtactggtgacgaaactcagtgttacggtacatgggttcctattgggcttgctatccctgaaaatgagggtggtggctctg
agggtggcggttctgagggtggcggttctgagggtggcggtactaaacctcctgagtacggt

5. Binding verification assay: zif268 + DNA

All binding reactions were performed on ice with a minimum of one hour of incubation time. The original binding buffer (selected to optimize protein binding) contains 200 mM KCl (required for nanopore measurements), 5 mM $MgCl_2$, 100 μM $ZnCl_2$, 10 mM Tris-HCl, 10% glycerol w/v, and 1 mM βME. This was based on the binding conditions described in previous studies of zif268[1], and tested and slightly modified for compatibility with our nanopore sensor. EMSAs for short oligo substrates were performed using 8% PAGE in non-denaturing conditions, and EMSAs for 1000bp substrates were performed using 3.5% PAGE in nondenaturing conditions. Gels were visualized using either a fluorescent Atto-647 tag (50 bp oligo with one binding site, used for high salt EMSAs), or with SYBR Green I (all other samples), and were scanned with a BioRad laser gel scanner.

We first verified that both the fused zif268_GST and the cleaved zif268 proteins bound specifically to their target site using ~50 bp oligomers. The (+) 50 bp oligo contains two binding sites separated by 6 bp, while (-) 50 bp oligo contains none. The EMSA for these binding reactions is detailed in Figure S4.

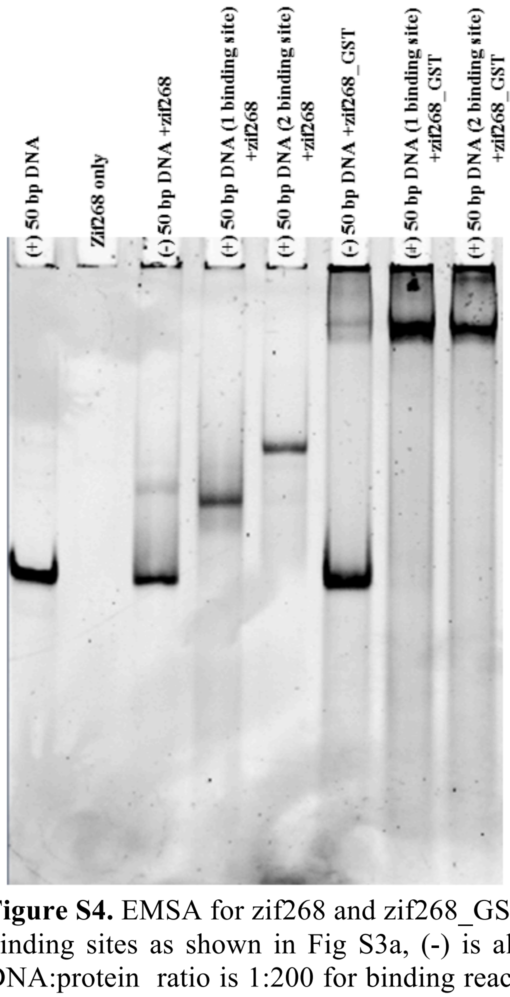

Figure S4. EMSA for zif268 and zif268_GST binding to short DNA oligos. (+) oligo is 50 bp with 1 or 2 binding sites as shown in Fig S3a, (-) is also 50 bp oligo with no binding sites as shown in Fig S3a. DNA:protein ratio is 1:200 for binding reaction with (+) 50 bp oligo with 1 binding site (lanes 4 and 7), 1:300 for (+)50 bp oligo with 2 binding sites (lanes 5 and 8).

Both zif268_GST and zif268 show specific binding to the oligos containing the consensus binding site(s), but do not bind to the negative control, which does not contain the consensus binding site. In addition, a higher band is observed in lane 5, indicating that the protein is binding to both potential binding sites. Note that the gel shift for bound zif268_GST is significantly larger than that for zif268 because of the large difference in protein molecular weight (37 vs. 10 kDa, respectively).

While the gel shift caused by zif268 and zif268_GST binding is readily apparent for small oligos, the shift is comparatively much smaller for binding to larger DNA. Figure S5 shows the gel shift for 1000 bp DNA with and without the consensus sequence for both proteins.

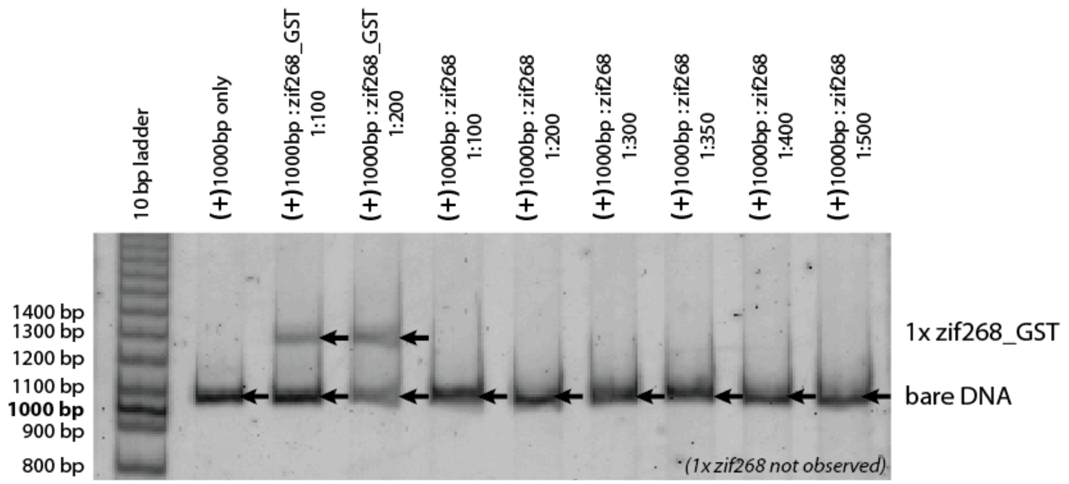

Figure S5: EMSA for zif268 and zif268_GST binding to 1000 bp DNA for nanopore. Note that the gel shift due to cleaved zif268 binding is not resolvable. This is consistent with its small size (10 kDa = 1.5% of 100 bp DNA mass) and low profile when specifically bound to DNA.

A clear mobility shift is visible for zif268_GST in Figure S5. We observe roughly 50% binding at a ratio of 1:250 (+)DNA:zif268_GST under these binding conditions, which is consistent with affinity levels observed by Elrod-Erickson and Pabo in their 1999 binding study[4]. However, there is no visible shift for the cleaved zif268. This is likely due to a combination of its low molecular weight (2% of the DNA molecular weight) and low profile binding site in the major groove of DNA.

Since the GST-tagged protein produces a clear shift, and both proteins were previously shown to exhibit similar binding affinity in shorter DNA, we assume that the zif268 also binds here. In subsequent EMSAs we infer binding of the smaller zif268 to long DNA by combining data from short oligo binding EMSAs using zif268 with long DNA binding EMSAs for the GST tagged version. This is confirmed by data obtained in a nanopore for the zif268-1000bp complex, as discussed in the main paper. For solid-state nanopore experiments it is preferable to work with high salt (~1 M KCl) buffers in the interest of both signal-to-background ratio and pore current

stability. We therefore checked the binding affinity of zif268 for its consensus binding sequence at high salt concentrations up to 1 M KCl. Figure S6 shows a clear mobility shift indicating binding of zif268 to the 50 bp fluorescently labeled oligo (single binding site) described above, even at salt concentrations up to 1 M KCl.

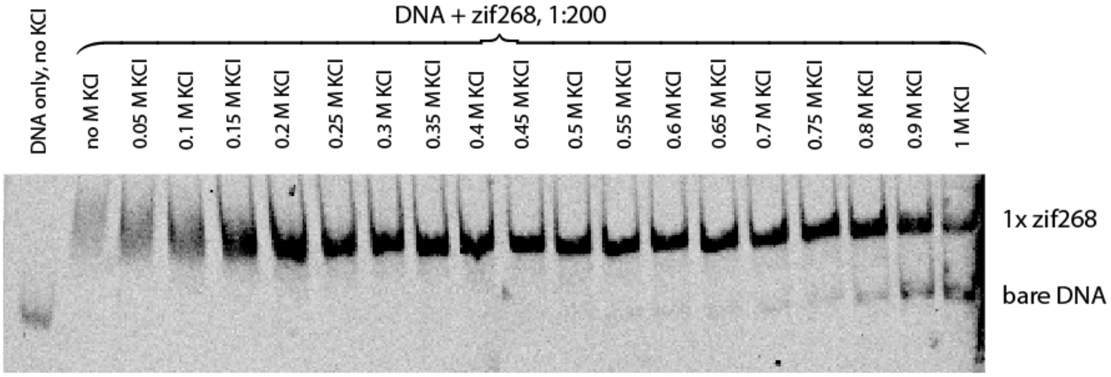

Figure S6: EMSA with salt gradient up to 1 M KCl for zif268-DNA binding. Binding ratio 1:200 DNA:zif268.

We also created a binding curve for zif268 and the same 50 bp Atto-647-labeled DNA at 1M KCl to determine what minimum working concentration could be used in a nanopore while still binding most of the DNA. From Figure S7 we see that no significant improvement to binding is gained above a binding ratio of 1:200 DNA:protein.

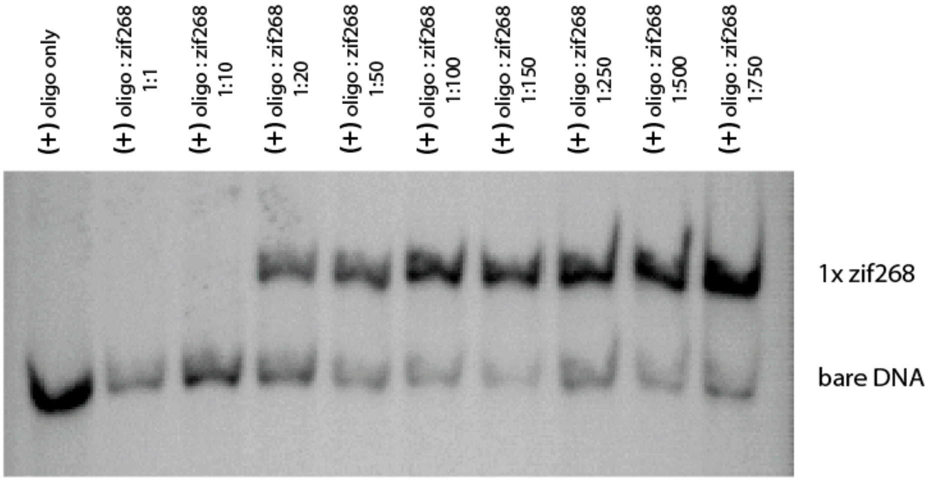

Figure S7: EMSA to detect binding of zif268/GST to DNA oligos.

6. Translocation events sublevel analysis

Blockage level patterns were determined by first identifying many possible blockage sublevels within an event using a change-point level-fining algorithm. This analysis produces multiple similar neighboring sublevels, which can be recombined into a simplified blockage level pattern with sublevels that fall into just a few blockage level ranges.

A recursive change-point algorithm based on maximum likelihood ratio was used to detect all blockage level change points in each translocation event that met a minimum likelihood threshold (typically 0.03-0.05). A sample reconstructed event based on this algorithm is shown in black in Figure S9a. The distribution of these blockage levels, shown in Figure S9b for a sample (+)DNA + zif268_GST data set, allows one or more major thresholds to be selected which separate the primary blockage states for the data set. These thresholds are used to combine neighboring sublevels (if they do not cross a threshold) to simplify the blockage pattern of the reconstructed event, recovering a blockage level and dwell time for each major sublevel within the event.

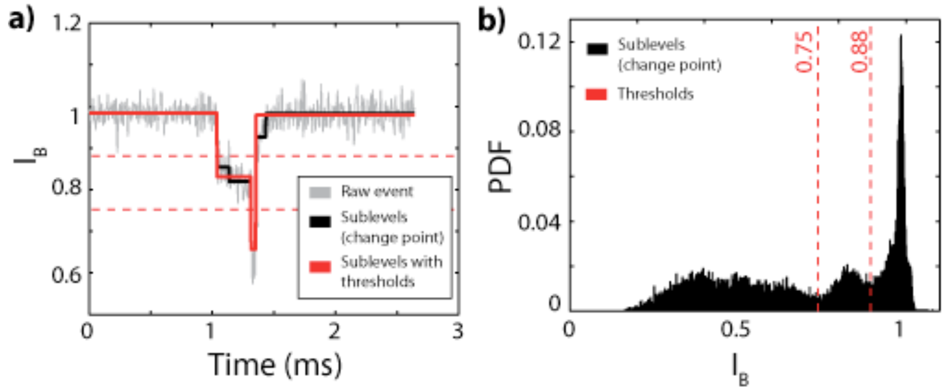

Figure S9: Sublevel detection and threshold analysis. a) Sample event (AB pattern) from (+)DNA with zif268_GST. Black line shows reconstruction of event from all initially detected sublevels (likelihood ratio threshold = 0.05). Red line shows reconstructed event after applying thresholds determined using histogram of all sublevels from all events. b) Blockage level histogram of all initially detected sublevels from all events in a dataset for (+)DNA with zif268_GST. Local minima are used to select thresholds to separate sublevel populations.

In this data set shown in Figure S9b, two major thresholds at $I_B = 0.88$ and $I_B = 0.75$ separate the sublevels into three clear populations: Open pore, A sublevel, and B sublevel. When neighboring sublevels are combined according to these thresholds, the final reconstructed event (red) will contain only sublevels whose neighbors are on the other side of one or more major

thresholds. For analysis of DNA+zif268_GST data, two thresholds were used to discriminate between open pore and A sublevel, and between the A and B sublevels, respectively. For analysis of DNA+zif268 data, a third threshold was necessary to further separate the B and C sublevels. Additionally, to facilitate identification of short sublevels which we here associate with specifically bound zif268, we found it effective to set the A□B major threshold as a fraction of the A blockage level (typically 1.2-1.5x of the A blockage level). Once the simplified translocation trace has been reconstructed, it is categorized according to its blockage pattern, for example open-A-open (“A”), open-A-B-A-open, etc.

7. Bound DNA + zif268_GST Translocations:

We began nanopore analysis of the DNA-TF complexes using the larger zif268_GST fusion protein. As evidenced by the event diagrams of all identified sublevels, shown in Figure S8, the blockage levels for bare (-)DNA (N=1280) in a 4 nm diameter pore differ from the blockage levels of both (-) and (+) 1000 bp DNA incubated with 1:200 zif268_GST (N=1488 and N=2155, respectively).

Translocations of (+)DNA and (-)DNA both with and without bound zif268_GST could be classified into three distinct categories of events, as shown in Figure S8: i) single level blockage at the same level as bare DNA (A sublevel only: (-)DNA+zif268_GST 58%, (+)DNA+zif268_GST 42%), ii) One additional sublevel occurring at the end of the event (AC sublevel pattern: (-)DNA+zif268_GST 27%, (+)DNA+zif268_GST 37%), and iii) Events consisting only of the deep sublevel (C sublevel only: (-)DNA+zif268_GST 6.5%, (+)DNA+zif268_GST 14%). All data sets were collected consecutively in a single 4 nm diameter nanopore at 400 mV bias relative to *cis*. Each point in the event diagrams represents a sublevel of the event rather than the full t_D or I_B of the event. Note that although there are only two sublevels for the blocked pore, we refer to them here as “A” and “C” (rather than “A” and “B”) because the deeper blocked level most frequently occurs at the end of the event and is relatively deep, similar to the AC patterns observed for zif268 without the GST tag.

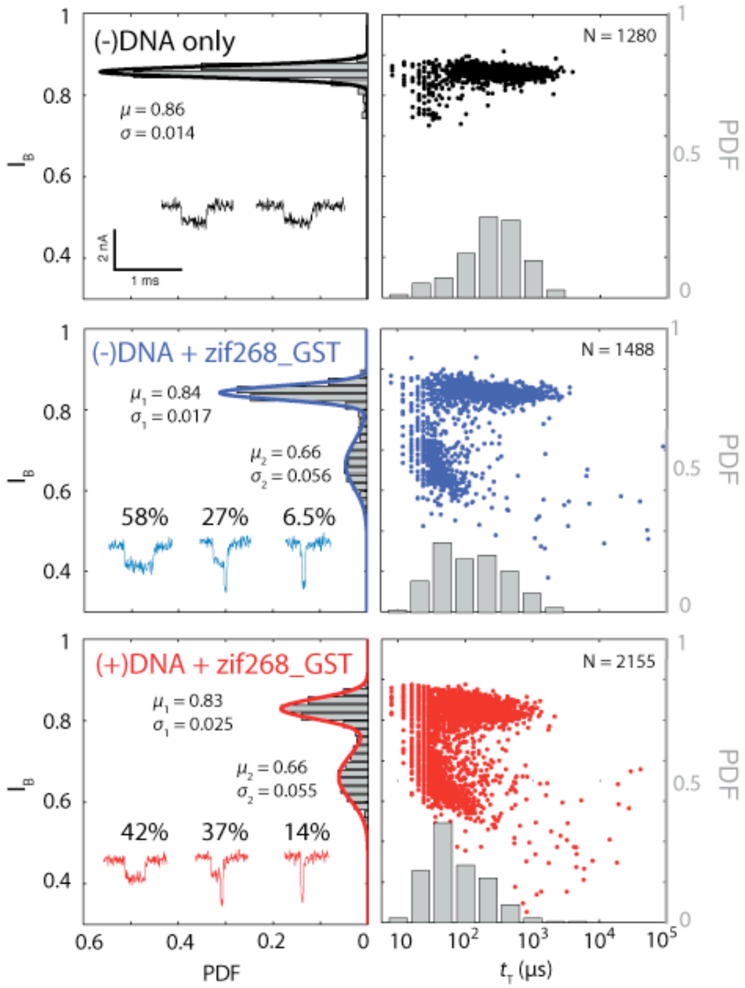

Figure S8: Event diagrams of all sublevels, blockage levels, translocation time, and event blockage patterns for DNA + zif268_GST translocations. Top: (-)DNA only (no zif268_GST). Middle: (-)DNA incubated 1:200 with zif268_GST prior to translocation shows additional deep blockage at the end of events. Bottom: (+)DNA incubated 200:1 with zif268_GST shows more frequent additional deep blockage levels relative to the (-)DNA+zif268_GST control.

Because the zif268_GST complex is too large to fit through the nanopore (zif268+DNA: 3 nm diameter, fusion GST tag adds 4-5 nm), the additional deep blockage level occurring at the end of events (AC blockage pattern) may be due to shear-induced rupture of the zif268_GST protein from its binding site or from a nonspecifically bound position on the DNA when it encounters the nanopore surface. Additionally, we note that the electrophoretic force applied on double-stranded DNA threaded in a pore can reach ~10^2 pN at 300 mV (depending on various variables, such as the effective charge of the DNA and interactions with the pore walls) which is comparable to the force required to rupture zif268 off of DNA as measured by AFM[5,6]. We note, however, that the rupture force depends strongly on the loading rate and directionality, which remain unknown for the nanopore system.

8. Additional data for DNA + zif268 translocations

Figure S9 shows data for bare (+)DNA, (-)DNA+zif268, and (+)DNA+zif268, all taken consecutively in a single nanopore (3.4 nm diameter, 300 mV applied bias). These data sets also directly show that the B level is only rarely observed on (-)DNA with zif268, and frequently observed on (+)DNA with zif268 (specific binding), while the C level is observed for both (+)DNA and (-)DNA in the presence of zif268 (nonspecific binding).

Figure S10 shows superimposed scatter plots from Figure S9 (bottom) for (-)DNA+zif268, and (+)DNA+zif268, illustrating that the A, B, and C levels maintain consistent blockage level and duration across all event patterns and also for the two different DNA samples. The density-colored scatter plots illustrate that A is the most common sublevel in both cases, and that the B level is more common for (+)DNA+zif268 than for (-)DNA+zif268.

In Figure S11, the duration of the B levels from ABA events and the C levels from AC events are examined for correlation with DNA level (total A level) duration. As shown in the main paper, the B levels for (+)DNA+zif268 are correlated with the DNA dwell time with high significance: (Pearson $\boldsymbol{\rho} = 0.47$ correlation, with significant $p = 6.1 \times 10^{-23}$) and are found roughly at the center of each event ($x_{rel} = 0.51$) while the C levels are not correlated with the DNA dwell time (Pearson $\boldsymbol{\rho} = 0.105$ correlation, with non-significant $p = 0.030$). The B levels for (-)DNA+zif268 are also correlated, albeit less strongly and with less significance than (+)DNA+zif268: (Pearson $\boldsymbol{\rho} = 0.33$ correlation, with significant $p = 0.0017$), and the C levels are also uncorrelated: (Pearson $\boldsymbol{\rho} = 0.025$ correlation, with non-significant $p = 0.66$).

Somewhat surprisingly, the x_{rel} for the (-)DNA+zif268 B levels is also well localized. However, the distribution is very different from that observed for (+)DNA+zif268, with two peaks instead of one, located at 0.41 and 0.79. This may indicate that there are one or more off-center sites of the (-)DNA+zif268 which have relatively high affinity for zif268. Candidate sites may be identified by peaks which are just under the threshold in Figure 3a (main paper).

Caption for Figure S9 (located on page 16)

Figure S9: Blockage level patterns for zif268+DNA in a thinned nanopore (3.4 nm diameter, 300 mV). Top: Sample translocation events showing the most common observed blockage level patterns: A, ABA, AC, ABAC, and C. Bottom: Scatter plots showing event average depth I_B and duration t_D (gray) as compared to sublevel depth I_B^i and duration t_D^i (by color, as indicated on inset sample events). a) (+)DNA only. b) (-)DNA + zif268 translocations. c) (+)DNA +_zif268 translocations. To facilitate visualization of population density, a random white noise offset below the acquisition rate of this data ($-2<\Delta t<+2$ μs, acquisition rate 250 kHz) has been added to the duration of each blockage level in all event diagrams.

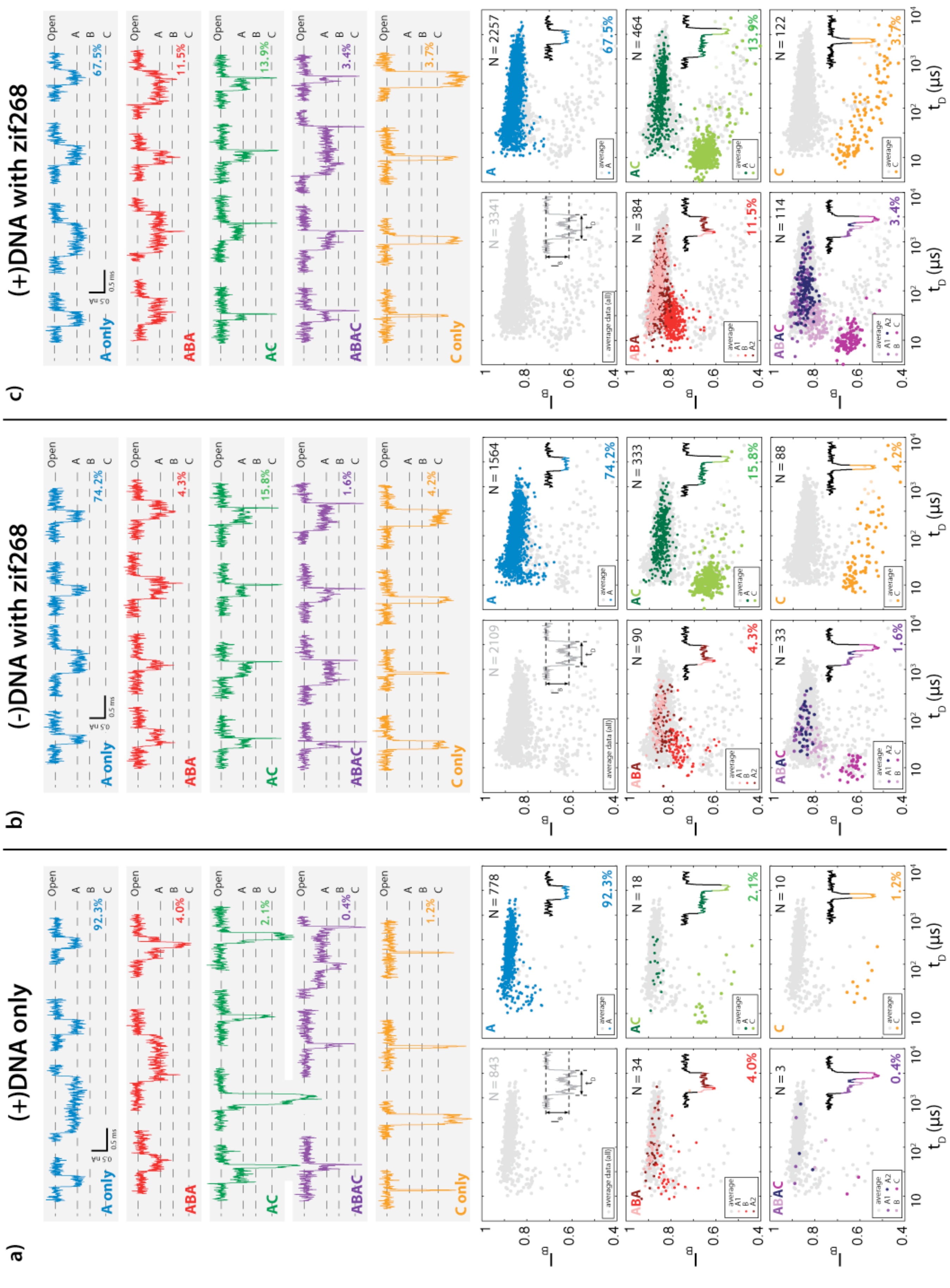

a)
(+)DNA only
b)
(-)DNA with zif268
c)
(+)DNA with zif268
A only
ABA
AC
ABAC
C only
Open
A
B
C
0.5 nA
0.5 ms
92.3%
4.0%
2.1%
0.4%
1.2%
74.2%
4.3%
15.8%
1.6%
4.2%
67.5%
11.5%
13.9%
3.4%
3.7%
N = 843
N = 778
N = 34
N = 18
N = 3
N = 10
N = 2109
N = 1564
N = 90
N = 333
N = 33
N = 88
N = 3341
N = 2257
N = 384
N = 464
N = 114
N = 122
average data (all)
average
A1
B
A2
C
I_B
t_D (μs)

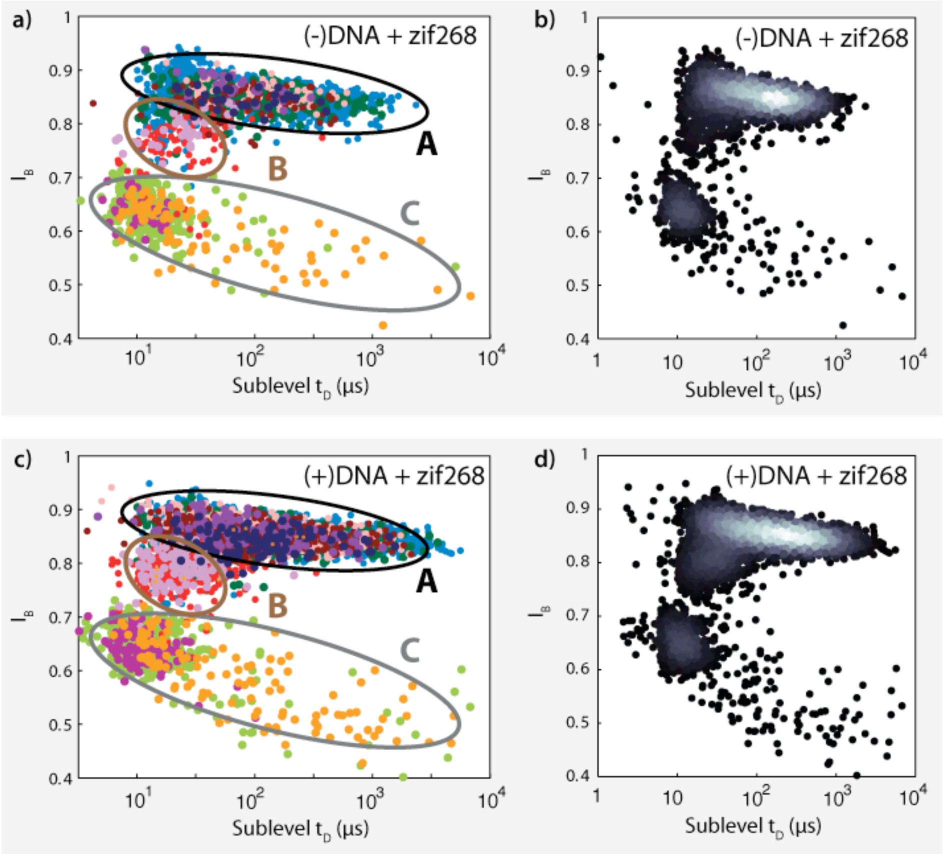

Figure S10: Cumulative A, B, and C sublevels from all categorized blockage patterns for both (-)DNA + zif268 translocations and (+)DNA + zif268 translocations. a) Superimposed scatter plot of A, B, and C levels from all blockage patterns for (-)DNA + zif268 translocations. Ellipses indicate A, B, and C populations. b) Scatter plot of all sublevels for (-)DNA + zif268 translocations, colored according to # of nearest neighbors to show local density (light = high density, dark = low density). c) Superimposed scatter plot of A, B, and C levels from all blockage patterns for (+)DNA + zif268 translocations. Ellipses indicate A, B, and C populations. d) Scatter plot of all sublevels for (+)DNA + zif268 translocations, colored according to # of nearest neighbors to show local density (light = high density, dark = low density).

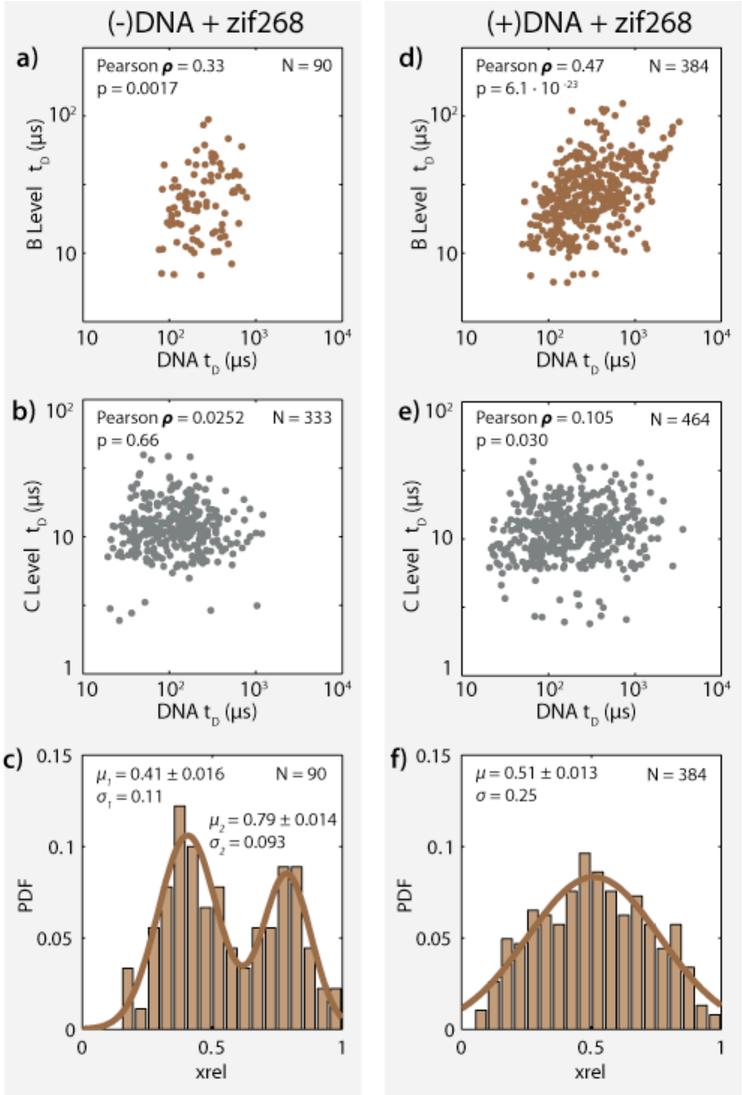

Figure S11: Timing of A, B, and C sublevels for (-)DNA + zif268 translocations and (+)DNA + zif268 translocations. a) Correlation of B level duration to total A level duration in ABA events for (-)DNA + zif268 (Pearson ***ρ*** = 0.33 correlation, with significant p = 0.0017). b) Correlation of C level duration to A level duration in AC events for (-)DNA + zif268 (Pearson ***ρ*** = 0.025 correlation, with non-significant p = 0.66). c) Distribution of observed relative position x_{rel} of the B level within ABA events with Gaussian fit for (-)DNA + zif268, N=90. d) Correlation of B level duration to total A level duration in ABA events for (+)DNA + zif268 (Pearson ***ρ*** = 0.47 correlation, with significant p = 6.1×10^{-23}). e) Correlation of C level duration to A level duration in AC events for (+)DNA + zif268 (Pearson ***ρ*** = 0.105 correlation, with non-significant p = 0.030). f) Distribution of observed relative position x_{rel} of the B level within ABA events with Gaussian fit for (-)DNA + zif268, N=384.

9. References

1. Christy, B. & Nathans, D. DNA binding site of the growth factor-inducible protein Zif268. *Proceedings of the National Academy of Sciences* **86**, 8737-8741, (1989).
2. Swirnoff, A. H. & Milbrandt, J. DNA-binding specificity of NGFI-A and related zinc finger transcription factors. *Molecular and Cellular Biology* **15**, 2275-2287, (1995).
3. Pfenning, A. R., Schwartz, R. & Barth, A. L. A comparative genomics approach to identifying the plasticity transcriptome. *BMC Neuroscience* **8**, 20-20, (2007).
4. Elrod-Erickson, M. & Pabo, C. O. Binding Studies with Mutants of Zif268: Contribution of Individual Side Chains to Binding Affinity and Specificity in the Zif268 Zinc Finger-DNA Complex. *Journal of Biological Chemistry* **274**, 19281-19285, (1999).
5. Wang, Y. *et al.* Analysis of DNA and Zinc finger interactions using mechanical force spectroscopy. *Nanobiotechnol* **2**, 87-93, (2006).
6. Dudko, O. K., Mathé, J., Szabo, A., Meller, A. & Hummer, G. Extracting Kinetics from Single-Molecule Force Spectroscopy: Nanopore Unzipping of DNA Hairpins. *Biophysical Journal* **92**, 4188-4195, (2007).
